# Glucose Can Protect Membranes against Dehydration Damage by Inducing a Glassy Membrane State at Low Hydrations

**DOI:** 10.3390/membranes9010015

**Published:** 2019-01-15

**Authors:** Alexander Dhaliwal, Adree Khondker, Richard Alsop, Maikel C. Rheinstädter

**Affiliations:** Department of Physics and Astronomy, McMaster University, Hamilton, ON L8S 4M1, Canada; alex.dhaliwal@mail.utoronto.ca (A.D.); khondkea@mcmaster.ca (A.K.); alsoprj@mcmaster.ca (R.A.)

**Keywords:** glucose, lipid membranes, sugar-membrane interactions, X-ray diffraction, molecular dynamics simulations

## Abstract

The physical effects of small sugars on membranes have been studied for decades, primarily because of their membrane stabilization in cold or dehydrated environments. We studied the effects of up to 20 mol% glucose in bilayers made of 1,2-dimyristoyl-*sn*-glycero-3-phosphocholine (DMPC) at low hydration by combining X-ray diffraction and Molecular Dynamics (MD) simulations. In agreement with previous studies, we observe membrane thinning at low and membrane thickening at high sugar concentrations. Glucose was found to preferentially localize to the outer head region of phospholipid bilayers at all concentrations, and partitioning of sugar in the membranes was found to monotonically increase with increasing sugar concentration. While the number of gauche defects in the lipid acyl tails and the lipid packing in the presence of sugar resembled values of a fluid lipid bilayer, tail dynamics, as assessed by autocorrelation of the carbon atoms in the phospholipid tails, were slowed down significantly with increasing glucose content. Thus, our findings suggest that sugar leads to a a disordered, glassy state of the hydrophobic membrane core. The non-monotonic effect of glucose on membrane thickness was found to be an effect of fluidification at low concentrations and decreased interdigitation in the higher sugar concentration regime.

## 1. Introduction

Sugars are ubiquitous components in all living organisms. They serve in a variety of structural and functional roles, from primary metabolic precursors to important intercellular signalling tools. While there exist protein-mediated mechanisms by which sugars can be translocated across cellular membranes in eukaryotic organisms, the basic interaction between sugars and lipid bilayers influences a variety of membrane properties [1,2,3,4,5,6,7]. Interplay between monosaccharides and disaccharides with the lipid membrane affects phase transition temperatures [3,4,7,8], fluidity [3,7,9], osmotic pressure [10,11], membrane width [5,12,13,14,15], and retention of structure during dehydration conditions [1,16].

Much of the early research was motivated by findings from Crowe et al. that pointed to the inherent ability of sugars to stabilize membranes in dehydration events [1]. Anhydrobiotic organisms with the ability to survive severe cold or dehydration were found to accumulate sugars in high concentrations [1,17,18], and it was hypothesized that this provided membrane protection and stabilization to prevent cellular rupturing. These concepts were extended to industrial applications, making simple sugars particularly attractive as an additive in commercial products spanning the fields of agriculture to cosmetics to pharmaceuticals [19]. This inspired much research into the realm of membrane stabilization in dehydrated systems using sugars [3,7,9,14,16,20,21,22,23,24,25,26].

**Figure 1:**
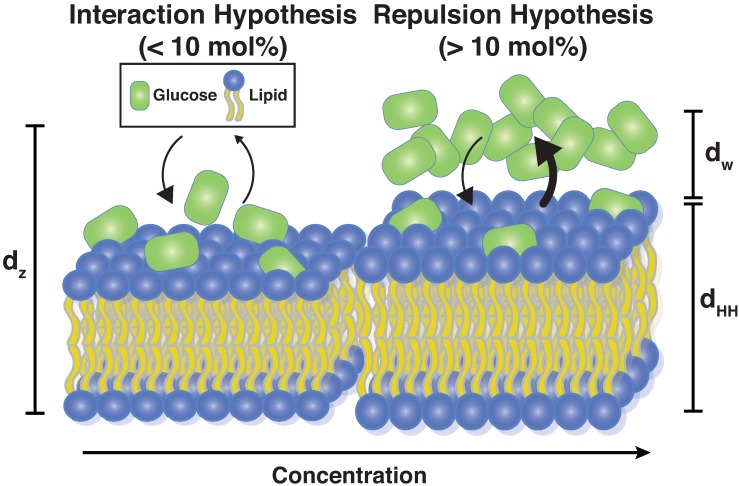
Current models of sugar-bilayer action suggests favorable, enthalpy-driven interactions dominate at lower sugar concentrations (the interaction hypothesis) while repulsive, entropy-driven effects dominate at higher concentrations (the repulsion hypothesis) [15]. $d_{z}$ denotes the lamellar membrane spacing, $d_{HH}$ the membrane thickness, and $d_{w}$ the thickness of the hydration water layer.

Two models emerged to describe the ability of sugars to stabilize membranes and alter their physical properties—the exclusion model and the interaction model, as pictured in Figure 1. The former suggested that sugars were preferentially excluded from the bilayer-water interface and remained within the bulk solution, indirectly increasing the interfacial free energy through osmotic imbalance that preserved the hydration shell surrounding phospholipid head groups [1,27,28,29]. In contrast, the interaction model posited that sugars substituted for water in the hydration shell of lipids, intercalating into the outer region of the bilayer through a network of hydrogen bonding [4,12,30]. In 2011, Andersen et al. presented an attempt to unify the two theories by providing evidence that favorable, enthalpy-driven interactions between sugars and phospholipid head groups dominated at low sugar concentrations and that repulsive, entropy-driven effects are most relevant at high concentrations [15].

We studied the interaction between glucose and dehydrated lipid membranes by combining X-ray diffraction and MD simulations. We were particularly interested in the molecular mechanisms behind the non-monotonic effects on certain physical properties of the membranes with increasing sugar concentration. We found that sugars localize to the interfacial head group region of phospholipid bilayers in a concentration-dependent manner. Glucose concentration was correlated with both increased tail disorder mimicking a fluid-like structure but at the same time decreased lipid tail dynamics.

## 2. Materials and Methods

### 2.1. Preparation of Multi-Lamellar, Solid Supported Membranes

Highly-oriented, multi-lamellar artificial lipid bilayers were constructed on 1 cm × 1 cm silicon wafers. The wafers were rendered hydrophilic and cleaned of organic residues using pre-treatment with dichloromethane (DCM) in a sonicating bath at 310 K. When removed from DCM, the wafers were cleaned by alternating 50 mL washes with methanol and ultra pure water (resistivity of 18.2 MΩ·cm), dried with nitrogen gas, and placed on an orbital shaker at 315 K.

1,2-Dimyristoyl-*sn*-glycero-3-phosphocholine (DMPC, Avanti Polar Lipids) was dissolved in a 1:1 mixture of 2,2,2-trifluoroethanol (TFE) and chloroform at an initial concentration of 18 mg/mL. Glucose (Sigma Aldrich) was dissolved at a concentration of 30 mg/mL in heated methanol at 320 K on an orbital shaker for 48 h, adapted from the protocol provided in [31]. The sugar solution was then diluted to a concentration of 3 mg/mL in excess TFE:chloroform.

Solutions of increasing glucose concentration were created through successive addition of a specific volume of the 3 mg/mL glucose solution to the lipid solution to create mixtures of the desired molar percentage of glucose. Use of this technique causes the DMPC concentration to decrease slightly with increasing glucose concentration, with the 20 mol% system having a DMPC concentration of 12.9 g/mL.

For each sample, 80 μL of sugar-lipid solution was applied onto a clean wafer and allowed to dry for 5 min on an orbital shaker at 320 K. Samples were placed in a vacuum chamber for 24 h to evaporate any remaining solvent. Samples were placed in an annealing chamber containing an open vial of ultrapure water and allowed to equilibrate to 293 K. The temperature was gradually increased to 303 K over 24 h. This procedure results in ordered, multi-lamellar membranes stacks of ∼3000 bilayers that uniformly cover the silicon substrate. Quality of all membrane samples was checked using an optical microscope and only samples with uniform lipid coverage were used for the X-ray experiments. The number of bilayers on each silicon chip can be estimated from the amount of material used during the preparation by knowing the number of molecules, the average area per molecule, and the area of the chip. By careful analysis of the reflectivity data in terms of absorption and peak width, the layer thickness can also be determined experimentally, and these values agreed well with the calculated thicknesses. A highly periodic structure is a prerequisite to obtain well-resolved out-of-plane and in-plane structural information using X-ray diffraction [32,33,34,35,36].

### 2.2. X-ray Diffraction

**Figure 2:**
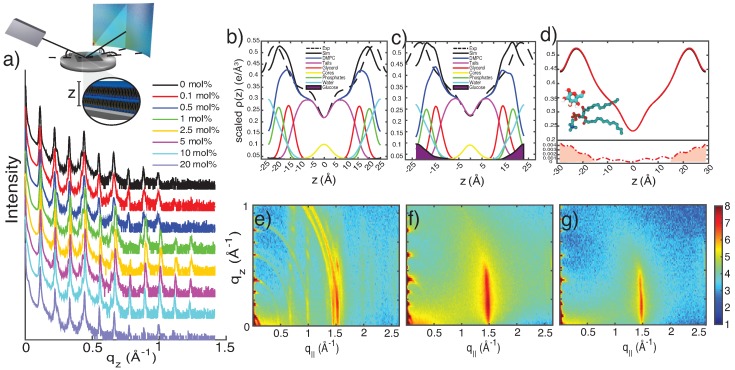
(**a**) Out-of-plane reflectivity data for DMPC bilayers with glucose concentrations from 0.1 mol% to 20 mol%. Inset shows a schematic of the experimental setup. (**b**,**c**) Comparison of electron density profiles (EDPs) from X-ray diffraction data (dashed black) and MD simulations (solid black) at 1 mol% (**b**) and 20 mol% (**c**) glucose. (**d**) Comparison of EDPs from X-ray diffraction data of a pure DMPC membrane and 1 mol% glucose. (**e**–**g**) Two-dimensional diffraction data for pure DMPC (**e**), 1mol% glucose (**f**), and 20 mol% glucose (**g**).

X-ray scattering data was collected using the Biological Large Angle Diffraction Experiment (BLADE) in the Laboratory for Membrane and Protein Dynamics at McMaster University. BLADE houses a 9 kW (45 kV, 200 mA) CuK*α* Rigaku Smartlab rotating anode that generates X-rays at a wavelength of 1.5418 Å with monochromatic intensities up to 10^10^ counts/(mm^2^·s). Both source and detector are mounted on moveable arms such that the membranes stay horizontal through a range of direct and oblique angles. Axial and planar periodicity of the samples allows for in-plane ($q_{\left|\right|}$) and out-of-plane ($q_{z}$) structures to be determined independently. Out-of-plane scans result in one-dimensional reciprocal space reflectivity plots, as seen in Figure 2a, while in-plane scans generate two-dimensional reciprocal space maps, as seen in Figure 2b–d. All scans were measured at 300 K in 50% relative humidity [37].

### 2.3. Calculating Electron Densities

The relative electron density, ρ(z), can be approximated as a 1-dimensional Fourier series:

(1) $$\begin{array}{ccc} \rho (z) & = & \frac{2}{d_{z}}\sum_{n=1}^{N}\sqrt{I_{n}q_{n}}\nu_{n}\cos\left(\frac{2\pi nz}{d_{z}}\right), \end{array}$$

where *N* is the highest order Bragg peak measured. The form factors, $F(q_{n})$, are the product of the integrated peak intensities, $I_{n}$, and their respective positions, $q_{n}$. Assuming the case of centro-symmetry, the bilayer form factor $F(q_{z})$ is real-valued. This simplifies the problem of phase determination to one of signs, wherein $F\left(q_{z}\right)=\nu_{n}\left|F\left(q_{z}\right)\right|$ and $v_{n}=\pm 1$. In order to determine the phase array required to reconstruct the electron density profiles as detailed in Equation (1), an analytical expression for the following continuous function, $T(q_{z})$, can be fitted to the membrane form factor $F(q_{z})$ at multiple peak positions $q_{z}$, as shown

(2) $$T\left(q_{z}\right)=\sum_{n}\sqrt{I_{n}q_{n}}\mathrm{sinc}\left(\frac{1}{2}d_{z}q_{z}-\pi n\right).$$

The phase array $v_{n}=\left[-1\;-1\;\;1\;-1\;\;1\;-1\;\;1\;-1\;\;1\;-1\;\;1\right]$ was found to fit optimally and was used for all samples.

### 2.4. Molecular Dynamics Simulations

Simulations were run on MacSim, a GPU-accelerated workstation with 20 physical Intel XeonCPU cores and two GeForce GTX 1080 graphics cards, totalling to 5120 CUDA cores. Standard 128 lipid membrane patches at low hydration can be simulated at a rate of approximately 200 ns per day using this setup. A system containing 128 DMPC lipids evenly sectioned across each leaflet was borrowed from Tieleman et al. [38]. The system was equilibrated at low hydration (7 waters per lipid) for 500 ns before glucose was added in varying concentrations. Glucose topology was manually validated by Lins and Hünenberger [39] using quantum mechanical optimization at the B3LYP/6-31G* level of theory [40]. The system was solvated using the SPC water model [41]. All simulations were performed using the GROMACS 5.1.2 software package [42,43], utilizing a GROMOS 54A7 force field [44] adjusted with Berger lipid parameters [45]. All simulations used a 2 fs time step, periodic boundary conditions applied in all directions, and a short-range van der Waals and electrostatic cutoff of 1.2 nm. The particle-mesh Ewald solution was used to calculate long-range electrostatics using a 0.16 nm grid spacing and cubic interpolation [46]. The LINCS algorithm was used for determination of all bond constraints [47]. Neighbor searching was performed on a grid with a short-range cutoff of 1.2 nm and a 10 fs update interval. A Nose-Hoover thermostat at 27 °C (time constant $\tau_{t}$ = 0.5 ps) was used for temperature coupling [48], and a Parrinello-Rahman semi-isotropic weak pressure coupling scheme was used to maintain a pressure of 1.0 bar (time constant $\tau_{p}$ = 1 ps, isothermal compressibility = 4.5 × $10^{-5}$ bar^−1^ axially and laterally) [49]. A topological overview of each simulated system is listed in Table 1.

A total of 16 simulations were conducted and analyzed—eight at 300 K and eight at 280 K. At 300 K, lipid systems containing 7 waters per lipid and 0 mol%, 0.8 mol%, 1 mol%, 2.5 mol%, 5 mol%, 10 mol%, and 20 mol% of β-glucose were constructed from a low hydration DMPC system equilibrated at 300 K for 500 ns. Water was removed from the system and glucose was added to the area outside of the bilayer using a Monte Carlo method. Systems were resolvated at 7 waters per lipid and simulated for 200 ns each, the final 50 ns of which were used for analysis. Glucose began interacting with the bilayer almost immediately after beginning the simulation. The choice of 7 waters per lipid derives from careful analysis of X-ray diffraction data of analogous DMPC systems containing glucose to mimic experimental condition. A hydrated 0 mol% system was also constructed by resolvating the DMPC bilayer with 27 waters per lipid, equilibrating for 50 ns, and then simulating for 200 ns.

The simulations conducted at 280 K were done to achieve a true gel-phase. To produce a gel-phase, the carbon bonds along the lipid chains were forced into an all-trans configuration and run for 100 ns, after which the bond constraints were released and the system was further equilibrated for 100 ns. From this point, all eight systems were created in an identical manner and composition as the 300 K simulations, and they were all run for 200 ns. All analyses showcased in the paper concern the 300 K simulations.

### 2.5. Molecular Dynamics Analysis

Electron density profiles were calculated for different constituents over the final 50 ns of each simulation, similar to other studies investigating lipid bilayers [37,50]. The calculation involves determining the relative distance along the bilayer normal of each atom within the defined index group, assigning a weight based upon the number of electrons corresponding to each atom, and producing an electron density curve as these values are averaged over the specified time range. The united-atom model of DMPC from these simulations uses a weighting of eight electrons for methylene carbons in the SN1 and SN2 phospholipid tails and a weighting of nine electrons for the terminal methyl of each tail. The results are displayed for each constituent in Figure 2e,f and in the Supplementary Material Figure S1. Membrane width, $d_{HH}$, was calculated using the peak-to-peak distance of the corresponding electron density profile. The hydration water layer thickness, $d_{w}$, was calculated as the difference of $d_{HH}$ from the average length of the axial dimension of the full-precision simulation data. Errors were estimated using block averages over five blocks [51].

As a measure of fluidity, the proportion of gauche dihedrals within the lipid tails were calculated for both the SN1 and SN2 chains. This parameter can be used to signify the degree of disorder within a bilayer system, with higher gauche proportions indicating a more fluid-like structure [36,52,53,54]. The fraction of gauche dihedrals was determined as a function of increasing distance from glucose. A script was written to create an index file containing dihedral sets of carbon chains belonging to lipids within the specified radius from the center of mass of any glucose molecule within the system every 25 frames (50 fs). The Ryckaert-Bellemans dihedral angles of these carbon sets (composed of windows of four carbons each down the length of the lipid tail) were calculated over that time interval [55]. This procedure was repeated for the final 50 ns of the simulations, and averages were taken for each carbon window of each system. For ease of visualization and interpretability, further averaging across the SN1 and SN2 tails generated the data points shown in Figure 4a. Successive analyses were performed for each new distance to glucose. Internal testing showed that using indices of dihedral sets shorter than 50 fs did not produce statistically different results, so this frame length was chosen to decrease computational time. Error bars were generated using the standard error of the data, calculated by dividing the standard deviation of the gauche fraction for each frame by the number of data points used.

Hydrogen bonding between water and lipid head groups as a function of increasing distance from glucose was also calculated by utilizing dynamic scripting and GROMACS algorithms. Distance and angle cutoffs for hydrogen bond classification were 3.5 Å and 30°, respectively. A script was written to produce an index file containing both lipid head groups (defined as the choline and phosphate moieties) within a certain radius of the center of mass of any glucose molecule in the system and the entire solvent group. The number of hydrogen bonds between these two groups and an index of participating lipids were calculated within the system every 50 frames (100 fs). This tactic was replicated for the final 50 ns of the simulation and for a series of distances from glucose. Averages across the simulation window were taken for each distance series, and the resulting values were normalized by the number of lipids participating at each frame considered. Error bars were generated in the same manner as those used in the gauche dihedral calculation.

Radial distribution functions were calculated to observe the relative spacing between various elements within the simulated systems. These functions, $g_{AB}\left(r\right)$, describe the probability of finding a designated particle or atom type *B* at a distance *r* from a reference particle or atom type *A*. Mathematically, they are formulated as the following:

(3) $$g_{AB}\left(r\right)=\frac{\langle \rho_{B}\left(r\right)\rangle }{{\langle \rho_{B}\rangle }_{local}}=\frac{1}{{\langle \rho_{B}\rangle }_{local}}\frac{1}{N_{A}}\sum_{i\in A}^{N_{A}}\sum_{j\in B}^{N_{B}}\frac{\delta (r_{ij}-r)}{4\pi r^{2}},$$

where $\langle \rho_{B}\left(r\right)\rangle$ is the probability density of particle *B* at a radial distance *r* from particle *A* and ${\langle \rho_{B}\rangle }_{local}$ is the average density of particle *B* over all spheres about *A* up to those with a radius equal to half of the minimum box length, used to normalize the value. $N_{A}$ and $N_{B}$ are the number of particles of type *A* and *B*, respectively. $\delta (r_{ij}-R)$ is the idealized δ-function that attains value at radii where particles of type *B* are located; however, the analytical technique used in this calculation simply divides the system in spherical shells of thickness 2 pm and makes a histogram across the total radial distance.

To assess intermolecular lipid spacing, radial distribution functions were calculated using the phosphorous atom in the lipid head groups as both the reference and the particle of interest. This allowed for the observation of how overall lipid headgroup packing structure varied due to the presence of glucose in varying concentrations.

As a gross comparison, the value of the phosphorous-to-phosphorous radial distribution functions at two distances were compared—0.45 nm, corresponding to the first and largest peak for the dehydrated DMPC system with 0 mol% glucose, and 0.55 nm, the first and largest peak for the hydrated DMPC system with 0 mol% glucose. Subtracting the latter from the former produced a comparative measure of hydrated to dehydrated lipid packing. The error bars were estimated by taking the difference between maximum and minimum values of the function within a six data point window about the peak position.

To assess lipid tail motion, the distance from the head group phosphate of each lipid to each carbon sequentially down the length of the SN1 and SN2 chains was calculated. An average was taken for these distances across all lipids, and the associated standard deviation of these lengths was used as a measure of relative motion between the tail and the head groups, i.e., higher standard deviations indicating a great range of motion. The absolute distances themselves were not found to correlate to system properties. This is because the strict measure does not directly indicate a more or less fluid structure, as it does not account for the relative angle between these theoretical vectors and the bilayer normal.

Lateral movement of lipids within the system was described using the calculated diffusion constants, denoted as $D_{xy}$ [53]. For the lipids, this was calculated by evaluating the mean square displacement (MSD) of the centre of mass of the head groups as projected onto the xy-plane perpendicular to the bilayer normal and averaging the result. The generalized form of the calculation is shown below:

(4) $$\lim_{t\to \infty }\langle ∥r_{i}\left(t\right)-r_{i}\left(0\right){∥}^{2}\rangle =4D_{xy}t,$$

where the Einstein relation generalized to two-dimensions was used to solve for $D_{xy}$ [56]. A fit via linear regression was taken over the time interval of 20 to 160 ns to calculate the diffusion constant, as these corresponded to the most linear portion of the MSD curves. Results are shown in the Supplementary Material Figure S2. The calculated values correspond well to DMPC systems at the conditions used [57].

As a further measure of lipid motion and a proxy for lipid phase, the rotational correlation function for lipid tails was calculated for the final 50 ns of each simulation. This was done by assessing the autocorrelation between the cross product of two vectors as defined by each tail carbon and the two carbon atoms adjacently bonded to it. Functions were calculated individually for each carbon window and averaged across the last 50 ns of simulation time. The function was fit to a first order Legendre polynomial before comparison. A generalized form of the autocorrelation function used is shown below [58]:

(5) $$C_{f}\left(t\right)={\langle f\left(\xi \right)f\left(\xi +t\right)\rangle }_{\xi },$$

where ξ is the time origin and f(t) is the time-varying property of interest. In this case, this is the direction of the cross product vector. Numerical integration of this function over 50 ns used was used to generate the rotational correlation time for each index. The incoherent nature of this calculation (observing the variation in these cross-products for individual lipids rather than a global average) allows for the probing of dynamic characteristics inaccessible to conventional experimental relaxation time analyses such as those used in neutron scattering experiments; as a result, certain molecular phenomena may become apparent through this analysis that are hidden in global average measures. Error bars are calculated as the standard deviation across the corresponding carbon windows of all bilayer lipids.

## 3. Results

The effect of glucose on lipid membranes was studied experimentally using highly-oriented, multi-lamellar lipid bilayers made of 1,2-dimyristoyl-*sn*-glycero-3-phosphocholine (DMPC). Membranes were prepared on silicon wafers for X-ray diffraction experiments, and analogous systems were constructed in silico based on experimental parameters, such that results between simulations and experiments could be compared directly. Full details regarding system preparation and subsequent analyses can be found in the Materials and Methods Section.

Glucose was added to both experimental and simulation assays in concentrations ranging from 0.1 to 20 mol%. Membranes were prepared and dehydrated for the experiments with ∼7 water molecules per lipid molecule in 50% relative humidity (RH). The levels of hydration used in simulations were chosen to mimic experimental conditions.

### 3.1. X-ray Diffraction

Out-of-plane diffraction is shown in Figure 2a. Between 8 and 11 Bragg peaks were observed, indicating well-ordered multi-lamellar membrane stacks. Peak intensity of higher order Bragg peaks was found to gradually decrease as a function of glucose concentration, indicative of an increasing degree of disorder.

To determine glucose’s position within the bilayer, electron density profiles (EDPs) were derived from the out-of-plane diffraction using Fourier analysis (Figure 2b–d). The experimental EDPs are shown together with EDPs calculated from MD simulations for comparison. Contribution of the different membrane components are plotted for the calculated EDPs. All profiles display a characteristic bilayer shape, with a minimum at 0 Å, representing the lipid core, and two symmetric maxima at ∼17 Å, representing the electron-rich phospholipid head groups. Membranes containing glucose display an increase in density beyond |z|>15 Å in both simulated and experimental systems. While this can be seen directly for MD systems via the glucose component of the EDPs in Figure 2b,c, a difference curve is used to illustrate this difference for experimental data between pure DMPC and DMPC containing 1 mol% glucose in Figure 2d. Additional EDP profiles for 0, 2.5, 5 and 10 mol% are included in the Supplementary Material in Figure S1.

We note that there is a slight difference between ρ(z) of the experimental and MD results in the tail region of the bilayer. This is likely due to limitations of the GROMOS 54A7 force field in replicating membranes with gel character, leading to simulations that consistently predict higher lipid area values and lower membrane width values [59]. Considering the discrepancy, the difference is expected and does not detract from the result that localizes glucose to the outer bilayer.

The spacing between successive peaks within the out-of-plane reflectivity data was used to determine the lamellar spacing, $d_{z}$, between adjacent bilayers in the membrane stack, as listed in Table 2. The inclusion of glucose at small concentrations results in a decrease of $d_{z}$. At concentrations above 2.5 mol%, the spacing was found to monotonically increase up to 20 mol% glucose. The intermembrane spacing $d_{z}$ was deconstructed into the membrane width, $d_{HH}$, as obtained by the peak-to-peak distance between the EDPs, and the water layer width, $d_{w}$. Values are included in Table 2, together with values from MD simulations.

In-plane diffraction for pure DMPC, DMPC with 1 mol% glucose, and DMPC with 20 mol% glucose is shown in Figure 2e–g. The multiple in-plane peaks seen in Figure 2e are typical for DMPC in its well-ordered gel phase (see for instance [33] for details). These features are not observed in Figure 2f,g, suggesting decreased gel character and a more disordered structure. The location of the most prominent in-plane peak at 1.5 Å^−1^ can be used to calculate the area per lipid chain, assuming dense packing with hexagonal symmetry, as reported for DMPC from neutron diffraction studies [60]. The area per lipid is chain determined as $a_{L}=8\pi^{2}/\left(\sqrt{3}q_{T}^{2}\right)$ [33,60,61], and calculates to 20.25 Å^2^ for all systems, in good agreement with literature values [13].

To evaluate the orientation of lipids in the sugar-lipid systems, the in-plane data around the lipid tail peak was integrated between 0.8 Å^−1^ < $q_{\left|\right|}$ < 2.6 Å^−1^ along the azimuthal angle from $10^{{}^{\circ}}<\phi <34^{{}^{\circ}}$ (smaller angles were omitted to avoid the effects of absorption). As a measure of orientation, Herman’s orientation function, $H=\frac{3}{2}<cos^{2}\left(\phi \right)>-\frac{1}{2}$, was fitted to the data. This function evaluates to H=1 for perfectly oriented bilayers and to H=0.25 for a randomly ordered isotropic system. Values for the 0 mol%, 1 mol%, and 20 mol% systems were found to be 0.968, 0.805, and 0.830, respectively, indicating less well oriented membranes in the presence of glucose.

### 3.2. Molecular Dynamics Simulations

**Figure 3:**
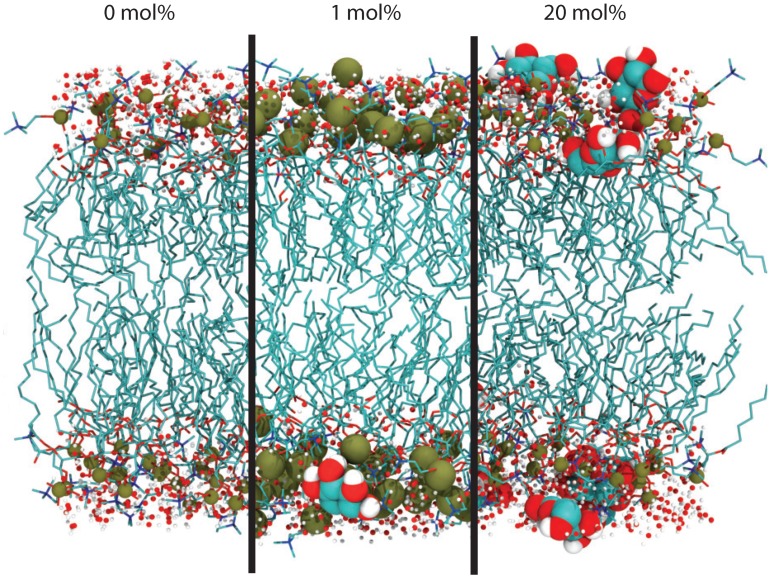
Comparison between dehydrated DMPC bilayers at 300 K for pure DMPC, 1 mol% glucose (enlarged phosphates), and 20 mol% glucose. Oxygen is displayed in red, carbon in teal, hydrogen in white, nitrogen in blue, and phosphorous in tan. Glucose spontaneously partitioned in the head group region of the membranes.

In order to compare dehydrated and hydrated membrane states, two systems were simulated: one with 27 waters per lipid (representing a fully-hydrated bilayer) and one with 7 waters per lipid (representing the dehydrated state analogous to experiment). A total of eight simulations at 300 K were conducted. In addition, eight simulations at a lower temperature of 280 K were conducted, as will be discussed below. Each simulation covered a minimum of 200 ns, resulting in 3.2 μs of simulation time. Simulations snapshots are shown in Figure 3.

#### 3.2.1. Molecular Dynamics: Structural Analysis

**Figure 4:**
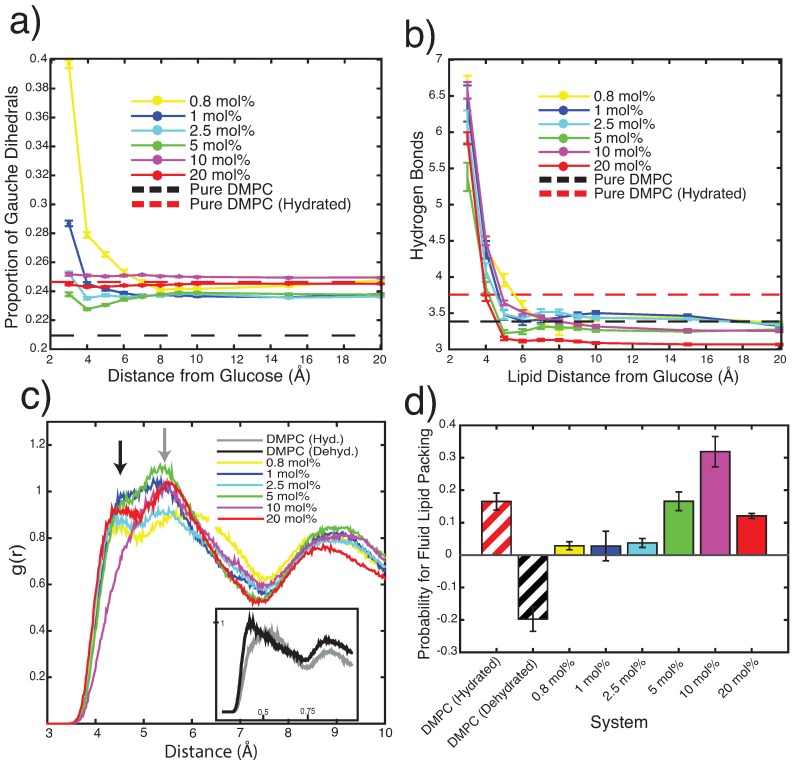
(**a**) Proportion of gauche dihedrals of SN1 and SN2 tails of DMPC at varying distances from glucose molecules. (**b**) Number of hydrogen bonds per lipid at varying radial distances from glucose. (**c**) Radial distribution functions for phosphate-to-phosphate distances in each lipid system. The dehydrated DMPC system exhibits a nearest phosphate peak at 4.5 Å, the hydrated DMPC system shows a broader peak at 5.5 Å. Sugar-lipid systems display a split peak between these values, indicating variation between hydrated and dehydrated lipid bilayer packing. (**d**) Difference between radial distribution function probability values at the hydrated peak position (5.5 Å) and the dehydrated peak position (4.5 Å). Positive differences indicate higher probability at the fluid position.

Structural features of the membranes were analyzed in different ways. To assess the ‘fluidity’ of the bilayers, the proportion of gauche defects was calculated as a function of distance from glucose within the bilayer, as shown in Figure 4a. Lipids closer to glucose displayed a higher number of gauche defects, indicating a more disordered structure. Dehydrated systems for all concentrations of glucose plateau at a stable dihedral proportion between 0.24 and 0.25. These values are in better agreement with those of hydrated DMPC bilayers ($0.2465\pm 7\times 10^{-4}$) than with dehydrated DMPC bilayers ($0.2094\pm 2\times 10^{-4}$).

Hydrogen bonding between water or glucose with lipid head groups as function of radial distance from glucose molecules is shown in Figure 4b. Hydrogen bonding between lipids immediately adjacent to glucose molecules in the bilayer was found to be greater (between 5.4 and 6.5 bonds per lipid) for all systems compared to lipids further away from the solute (between 3 and 3.5 bonds per lipid). However, the overall number of hydrogen bonds (represented as the data Figure 4b) at the furthest distance from glucose that, thus, considers the largest number of lipids) more closely resembles the bonding scheme of dehydrated bilayers than hydrated bilayers. This is supported by a decrease in lateral diffusion with sugar concentration (shown in the Supplementary Material Figure S2). So while the gauche defect density in the presence of glucose is comparable to a hydrated DMPC bilayer, hydrogen bonding pattern and diffusion are in agreement with dehydrated bilayers.

Radial distribution functions of the lipid phosphates were calculated to observe the effects of glucose concentration upon overall lipid packing, shown in Figure 4c. Dehydrated pure DMPC systems exhibit a first peak at a radial distance of 4.5 Å, while hydrated bilayers show a first peak at a slightly larger distance of 5.5 Å. Bilayers containing different amounts of sugar show split peaks centered at approximately these two radial distances but with different intensities of the two components. Figure 4d shows the difference in probability values from the radial distribution function for each system at 5.5 Å and 4.5 Å, where a positive difference shows increased similarity to the hydrated distribution peak location. All glucose membranes show positive differences, corresponding to a lipid packing distribution more similar to that of a hydrated system than a dehydrated system.

#### 3.2.2. Molecular Dynamics: Dynamical Analysis

**Figure 5:**
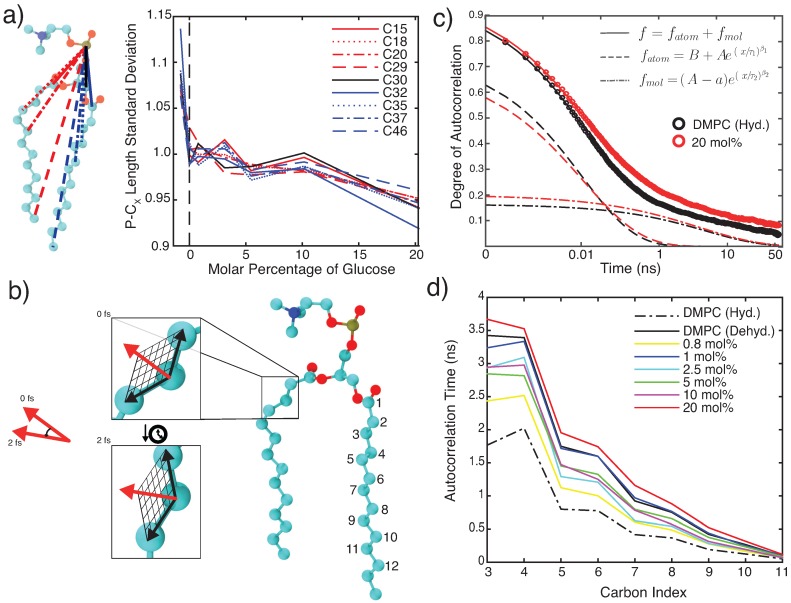
(**a**) Normalized standard deviations of the distances between phosphorous and various carbon atoms in the phospholipid tails as a function of glucose concentration. Blue curves use carbons from the SN2 tail, red curves use carbons from the SN1 tail, and black curves use carbons from the glycerol moiety. Data show a decrease in standard deviation with increasing sugar concentration, indicating a decrease of mobility. (**b**) Schematic showing how rotational autocorrelation was calculated. Windows of three adjacent carbon atoms on the lipid tails were selected, and the change in the vector normal to the plane generated by these three atoms was observed across 50 ns. Numbering convention of the carbon windows is shown. (**c**) Typical autocorrelation functions for 0 mol% hydrated system and 20 mol% dehydrated bilayers. Data was fitted using two stretched exponentials. (**d**) Autocorrelation time as a function of carbon window, calculated as the integral across the autocorrelation function.

Although gauche defects are often used as a measure of membrane fluidity, they describe a structural feature of a dynamic phenomenon. As a result, dynamic lipid information is required to truly conclude a fluid state. A more dynamical measure of increased fluidity is the fluctuations of intramolecular distances within each lipid molecule. The standard deviation of the distance between the lipid phosphate atom and select carbon atoms within the SN1 tail, glycerol moiety, and SN2 tail of DMPC is shown in Figure 5a. The first and last carbon atoms of each chain were chosen to best probe intramolecular motion at both the head-tail interface and bilayer core. Highest standard deviations were found for pure hydrated DMPC bilayers. Increasing glucose concentration was found to decrease distance fluctuations, suggesting that intramolecular lipid motions decrease as a function of glucose concentration.

Rotational autocorrelation functions along the lipid tails were calculated to provide a quantitative measure of lipid bilayer dynamics. Autocorrelation between the vector normal to the plane defined by adjacent carbons in a triplet window (as sketched in Figure 5b) was assessed. Results are shown in Figure 5c. Carbon triplet windows in the methylene region of the tail (windows 4–7 as shown in Figure 5b) were found to produce the most consistent data as various 50 ns time windows in the simulations were sampled for data analysis. The hydrated DMPC bilayers were found to decay faster when compared to bilayers containing 20 mol% glucose, shown in Figure 5c.

The autocorrelation time, i.e., the time after which a specific lipid tail position is no longer related to its initial orientation, was calculated as the integral beneath the functions, and is shown in Figure 5d. Data is shown for carbon windows 3–11 as an example, displaying a decrease with window number as we analyze carbon windows closer to the methyl terminus. These carbons possess more degrees of freedom within which tail restructuring can occur. The hydrated system has the shortest autocorrelation time at all points in the carbon tail. However, this difference becomes less pronounced closer to the bilayer core. In contrast, the 20 mol% glucose system has the longest autocorrelation time for all carbons along the acyl tail.

To further characterize these curves, the decay of the autocorrelation function was fit using the sum of two stretched exponential functions: $f=B+Ae^{{\left(t/\tau_{1}\right)}^{\beta_{1}}}+\left(A-a\right)e^{{\left(t/\tau_{2}\right)}^{\beta_{2}}}$. A good fit was obtained when the first exponential term had its time constant fitted between 1 and 100 ps, while the second term used a time constant between 0.1 and 10 ns. While the former tentatively describes motion on an atomic timescale, the term with the longer time constant is likely associated with larger-scale, molecular motions. All fits attained $R^{2}$ values greater than 0.99. Out of the 12 possible triplet carbon windows on each lipid tail, analysis was found to be most consistent when windows in the middle of the tail were chosen (indicated as 4–7 in Figure 5b).

**Figure 6:**
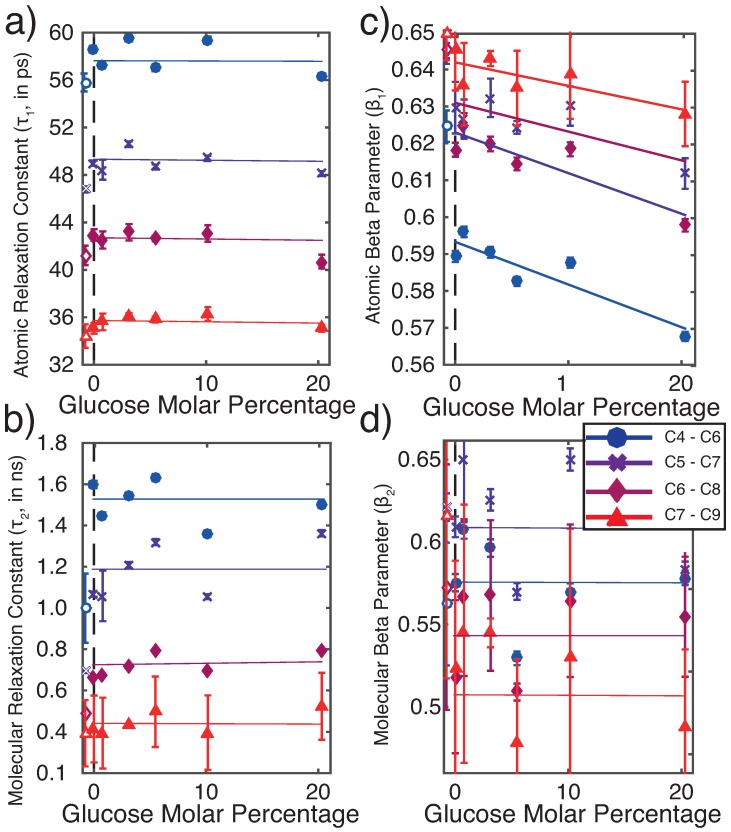
Parameters determined from fits of two stretched exponentials to the autocorrelation function. Windows 4–7 are displayed for analysis as these windows yielded least variable fits. Values below 0 with hollowed markers correspond to fully hydrated membrane system. Atomic time constant, $\tau_{1}$ (**a**), and molecular time constant, $\tau_{2}$ (**b**), are independent of glucose concentration and decrease towards the tails. (**c**) Atomic stretching exponent, $\beta_{1}$, decreases with sugar concentration and increases towards tails while the molecular stretching exponent, $\beta_{2}$, (**d**) is independent of both sugar concentration and carbon window.

Fitting results are shown in Figure 6. A table of all fitted parameters is included in the Supplementary Material Table S1. Plotted are the relaxation times, $\tau_{1}$ (a) and $\tau_{2}$ (b), and the corresponding stretching exponents, $\beta_{1}$ (c) and $\beta_{2}$ (d), as a function of glucose concentration for carbon windows 4–7. While the relaxation time constants and $\beta_{2}$ were found to be approximately constant across glucose concentrations, the time constants decrease for carbon windows closer to the ends of the lipid tails. In contrast, the stretching exponent $\beta_{1}$ displayed a clear negative dependence on glucose content. We tentatively interpret the atomic $\beta_{1}$ parameter as a measure of the ‘glassiness’ of the membrane core, as smaller stretching parameters are associated with more glass-like behavior [62]. This parameter decreases monotonically with glucose concentration and increases with carbon window number. The change with carbon window number shows increased interaction in tail regions closer to the head group, whereas the glassy nature of the membrane declines when areas closer to the methyl terminus are considered.

## 4. Discussion

Previous studies using a variety of techniques reported that sugar-membranes form with sugar located within the lipid head groups and in the aqueous region surrounding the bilayers [4,27,28,29,30]. In agreement with this literature, all bilayers in our study showed glucose localization to the bilayer-water interface for concentrations from 0.1 mol% to 20 mol%.

Experiments and simulations present evidence that the presence of glucose in dehydrated bilayers increases membrane tail disorder. In-plane X-ray diffraction, gauche defect density, and lipid packing resemble values typically found in hydrated, fluid lipid membranes. Tail dynamics measured by the autocorrelation function and autocorrelation time, however, were significantly slowed down and showed stretched exponential decays typical for slow, glassy dynamics.

We note that the pure DMPC simulations did not transition spontaneously into a true gel-phase at low hydration and 300 K, as suggested by the experimental results. Previous studies have shown an inability to construct a true gel-phase at 300 K for the GROMOS54a7 forcefield [59]. To compensate for this, additional simulations were run wherein the carbon bonds along the lipid chains were forced into an all-trans configuration for 100 ns, after which the bond constraints were released and the systems were run at 280 K. These simulations produced a gel-phase similar to that of pure DMPC from experimental results in terms of area per lipid and membrane width. All the sugar-lipid systems were also replicated and simulated for 200 ns. However, the sugar-lipid systems generated in this way did not transition into a disordered phase, as seen in experiment. This is likely due to the step in the experimental protocol wherein systems are fully hydrated during annealing. For the pure DMPC system at 280 K, the structural parameters analyzed (proportion of gauche dihedrals, hydrogen bonds, radial distribution function) all agree with the structural trends showcased in Figure 4. However, due to the heavy dependency of membrane parameters on temperature, differences in the absolute values were observed. We therefore argue that the 300 K systems in this study are more realistic and more relevant as they are closer to the experimentally prepared systems.

### 4.1. Glucose Slows Down Lipid Bilayer Dynamics

The time dependence of the autocorrelation function was best fitted by two stretched exponential functions, one with a long time constant and the other a short time constant. We tentatively assign these two processes to fast atomic relaxations and slower molecular reorientations (similar to β- and α-relaxations in glasses [62]). Physically, these correspond to picosecond and nanosecond timescales, which appropriately correlate to atomic motions such as bond oscillations (∼1 ps) or gauche-trans isomerization (∼100 ps) and molecular motions such as lipid protrusion (∼1 ns) and rotational diffusion (∼10 ns) [63]. These relaxation times were found to depend on the position along the lipid tail but appeared to be independent of glucose concentration.

The stretched exponential $\beta_{2}$ for the molecular term did not correlate with glucose, suggesting that correlation of one portion of the lipid tail is not dependent upon the motion of the entire lipid—intuitively, the presence of sugar should not promote this. However, the atomic exponent, $\beta_{1}$, displays a distinct decrease with sugar concentration, as well as a decrease when comparing the hydrated DMPC system to the dehydrated system. In stretched exponentials, smaller stretching exponents reduce the rate of decay; in a physical context, this implies greater interaction between elements in the system, akin to a more glassy, interconnected structure. The glassy character becomes less pronounced towards the centre of the bilayer (C-atoms with larger indices) due to increased distance from glucose and greater free volume.

### 4.2. Glucose Has a Non-Monotonic Effect on Bilayer Spacings

**Figure 7:**
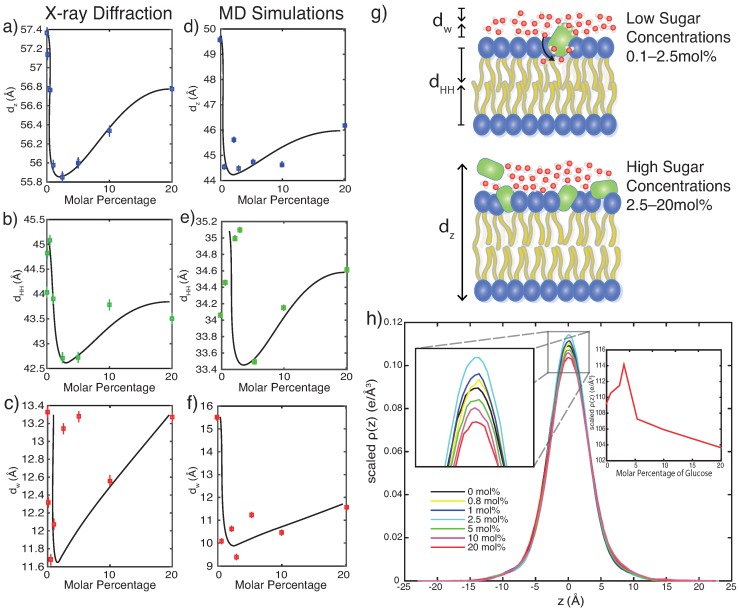
Bilayer thickness parameters from experiments (**a**–**c**) and simulations (**d**–**f**). Lamellar spacing $d_{z}$, membrane width $d_{HH}$, and water layer thickness $d_{w}$ are found to decrease sharply at low concentrations, and gradually increase towards higher glucose concentrations. (**g**) Schematic of non-monotonic behavior for bilayers at low and high glucose concentrations. (**h**) Electron density profiles for the core component (terminal methyl carbons of the phospholipid acyl chains) from MD simulations (from Figure 2b,c). The height of this peak is a measure of interdigitation, as it displays the relative probability of phospholipids occupying the centre of the bilayer. Insets show close-ups of the region between −2.5 Å <z< 2.5 Å. Increased interdigitation is seen at low glucose concentrations but decreases as glucose content increases.

The intermembrane spacing, $d_{z}$, the membrane width, $d_{HH}$, and the hydration water layer width, $d_{w}$, from experiments and simulations are plotted in Figure 7a–f and show the well-known non-monotonic behavior with increasing glucose concentration: a step decrease in width at low concentrations followed by a gradual increase towards higher concentrations. We note that at the same time the partitioning of glucose in the bilayers was found to be monotonically increasing with glucose concentration. In order to provide a molecular explanation for the non-monotonic behavior of the structural parameters, Figure 7h also shows the electron density in the center of the bilayers from the calculated EDPs in Figure 2b,c (the terminal methyl carbons of the phospholipid tails, the ‘Core’ component). This density is a direct measure of lipid tail interdigitation. This bilayer interdigitation followed the same non-monotonic behavior, mimicking the change in membrane thickness. The different regimes can be discussed separately:

Low concentrations (0.1 mol% to 2.5 mol%): All glucose molecules interact with the head group region at low concentrations, disrupting lipid packing and causing an increased water density in the head-tail interface. This leads to a decrease in hydration water layer thickness, and the increased fluidification significantly reduces bilayer width. As the membrane thins, lipid tail interdigitation sharply increases.

High concentrations (2.5 mol% to 20 mol%): As tail motions slow down, the degree of tail interdigitation monotonically decreases. This leads to an increased membrane width as compared to the more fluid-like structures at low sugar concentrations. At the highest sugar concentrations, glucose molecules have saturated the lipid head groups and start to reside also in the aqueous layer. As a hygroscopic molecule, this attracts water to its position, leading to an increase in both the width of the water layer and the overall spacing.

The non-monotonic behavior of the membranes’ structural parameters with increasing glucose concentration can thus be interpreted as a result of the monotonically increasing partitioning of glucose molecules in the bilayers.

## 5. Conclusions

We studied the interaction between sugar and dehydrated phospholipid bilayers made of DMPC and between 0.1 mol% and 20 mol% glucose using X-ray diffraction and MD simulations. Sugar molecules were found to monotonically partition in the head group region of the lipid bilayers with increasing glucose concentration. Experiments and simulations confirm the well-known behavior: a thinning of the membranes at low sugar concentrations and a thickening of the membranes at high sugar concentrations.

The presence of sugar in dehydrated membranes was found to lead to an increase in the gauche defect density of the lipid acyl tails and a loosening of the lipid packing, mimicking characteristics of disordered, fluid-like, hydrated membranes. However, tail motions as measured by the tail autocorrelation function were found to be significantly slowed down. Stretched exponential functions were fitted to the autocorrelation of different positions across the lipid acyl tail. Increasing glucose concentration led to a decrease of the stretched exponent, indicating glassy dynamics at high sugar concentrations.

This likely points to a bilayer vitrification phenomenon, whereby dehydrated membranes saturated with glucose adopt a low-motion, disordered structure. This would have important physical and biological implications, such as suppressed structural membrane transitions that would allow for the membrane freezing and increased stabilization observed in the event of severe dry or extreme cold conditions.

## Figures and Tables

**Table 1:** Overview of the systems prepared for the MD simulations. All simulations were run for at least 200 ns, and the last 50 ns were used for analysis. The number of hydration water molecules was chosen to be 7 for the dehydrated systems, in accordance with experimental evidence, making the experimental and MD systems directly comparable.

# DMPC Molecules	# Glucose Molecules	Water/Lipid (sim)	Glucose mol% (exp)	Run Time (ns)	Temperature (K)
128	0	27	0	200	300
128	0	7	0	500	300
–	–	–	0.1	–	300
–	–	–	0.5	–	300
128	1	7	1	200	300
128	3	7	1	200	300
128	4	7	2.5	200	300
128	7	7	5	200	300
128	13	7	10	200	300
128	26	7	20	200	300
128	0	27	–	200	280
128	0	7	0	200	280
128	1	7	1	200	280
128	3	7	1	200	280
128	4	7	2.5	200	280
128	7	7	5	200	280
128	13	7	10	200	280
128	26	7	20	200	280

**Table 2:** Summary of the experimental and simulated structural parameters. Given are the number of water molecules, $n_{w}$, the area per lipid molecule, $A_{L}$, lamellar spacing, $d_{z}$, membrane width, $d_{HH}$, and hydration water layer thickness, $d_{w}$. The area per lipid tail was determined from the experiments to be 20.25 Å^2^.

Glucose (mol%)	$n_{w}$	$A_{L}$ (Å^2^)	$d_{z}$ (Å)	$d_{\mathrm{HH}}$ (Å)	$d_{w}$ (Å)
X-ray diffraction					
0	7	–	57.4	44.0	13.3
0.1	7	–	57.1	44.8	12.3
0.5	7	–	56.8	45.1	11.7
1	7	–	56.0	43.9	12.1
2.5	7	–	55.9	42.7	13.1
5	7	–	56.0	42.7	13.3
10	7	–	56.3	43.8	12.6
20	7	–	56.8	43.5	13.3
MD simulations					
0	25	59.72±0.05	63.0	41.6	21.4
0	7	56.65±0.05	49.6	34.1	15.5
0.8	7	57.33±0.05	44.5	34.5	10.1
1	7	57.31±0.05	45.6	35.0	10.6
2.5	7	57.19±0.05	44.5	35.1	9.4
5	7	57.39±0.04	44.7	33.5	11.2
10	7	57.58±0.05	44.6	34.2	10.5
20	7	57.48±0.05	46.2	34.6	11.6

## Supplementary Materials

The following are available online at http://www.mdpi.com/2077-0375/9/1/15/s1. Figure S1: Electron Density Profiles for X-ray diffraction data (dashed black) and MD simulations (solid black) at (A) 0 mol%, (B) 2.5 mol%, (C) 5 mol%, and (D) 10 mol%. Experimental results and simulations correspond well to one another. Additional height of profile at methylene region between 5 Å < z < 12 Å is likely due to increased tail packing density in experiments, a known limitation of the GROMOS 54A7 forcefield used for simulations, Figure S2:Lateral diffusion constant for phospholipids as a function of molar percentage of glucose from MD simulations. The phosphorous atoms in the lipid headgroups were used to track diffusion and linear regression as the associated mean squared displacements were used to derive a diffusion coefficient. Results correspond well to similarly structured systems. Lipid diffusion peaks at intermediate glucose concentration before again decreasing, achieving values beneath that of a pure dehydrated DMPC bilayer at 20 mol%. The initial increase suggests that the presence of the solute disrupts lipid packing and leads to increased lateral movement at intermediate concentrations; however, at higher glucose levels, the impedence to movement resulting from the additional molecular presence slows down overall movement. This is supported by the hydrogen bonding data wherein the highest sugar concentrations display the lowest asymptotic number of hydrogen bonds per lipid, suggesting that the lipids are not optimally structured due to the increase solute presence. Diffusion constants for glucose molecules were found to be independent of concentration. Table S1. Parameters of double stretched exponentials used to fit autocorrelation functions for MD systems. All data sets were fit to an equation of the form $f=B+Ae^{{\left(t/\tau_{1}\right)}^{\beta_{1}}}+\left(A-a\right)e^{{\left(t/\tau_{2}\right)}^{\beta_{2}}}$. The four methylene carbon windows described here are on the SN1 chain of the DMPC lipids analyzed. All fits achieved an r^2^ value greater than 0.99.
