# Supplementary material for: Glucose Can Protect Membranes against Dehydration Damage by Inducing a Glassy Membrane State at Low Hydrations
Source: Membranes (Basel). 2019 Jan 15;9(1):15. doi: 10.3390/membranes9010015 (PMC6359629; doi:10.3390/membranes9010015)
Supplement: Supplementary file 1 [file membranes-09-00015-s001.pdf]

membranes

MDPI

Supplementary Materials: Glucose Can Protect Membranes against Dehydration Damage by Inducing a Glassy Membrane State at Low Hydrations

Alexander Dhaliwal, Adree Khondker, Richard Alsop and Maikel C. Rheinstädter *

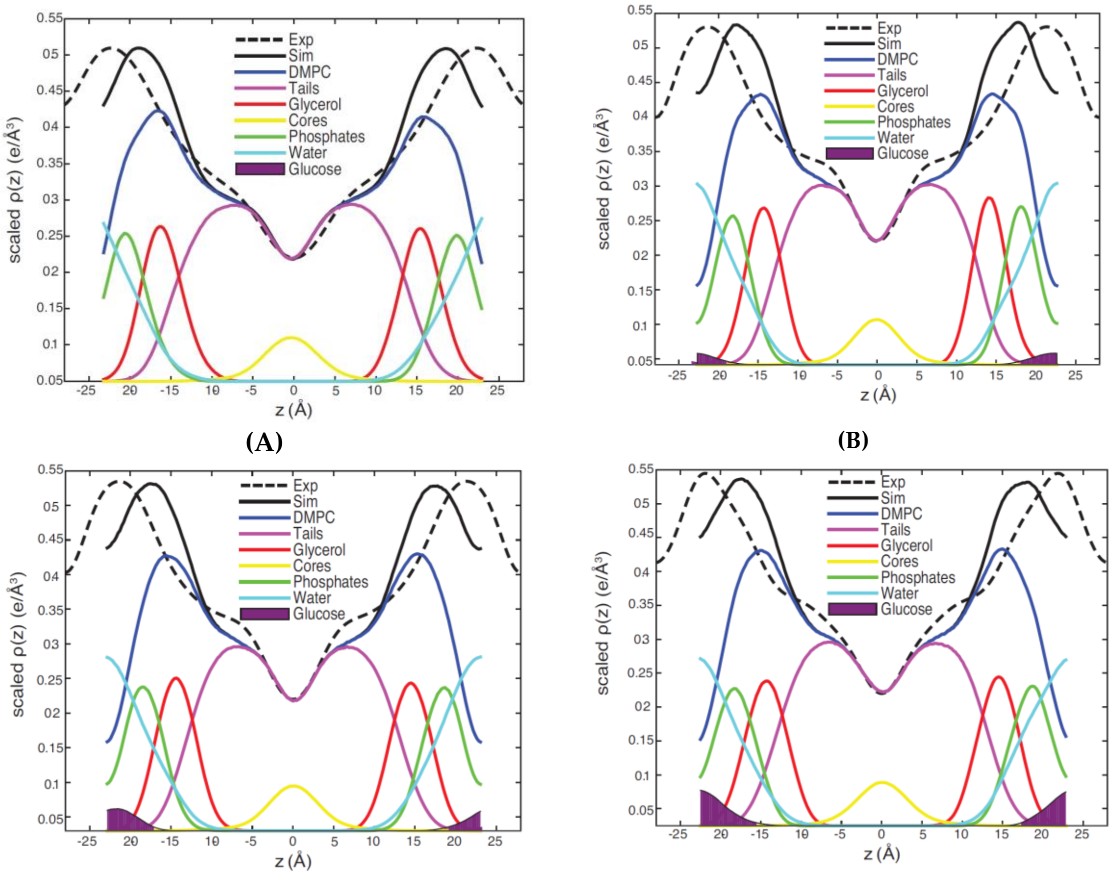

Figure S1. Electron Density Profiles for X-ray diffraction data (dashed black) and MD simulations (solid black) at (**A**) 0 mol%, (**B**) 2.5 mol%, (**C**) 5 mol%, and (**D**) 10 mol%. Experimental results and simulations correspond well to one another. Additional height of profile at methylene region between 5 Å < z < 12 Å is likely due to increased tail packing density in experiments, a known limitation of the GROMOS 54A7 forcefield used for simulations.

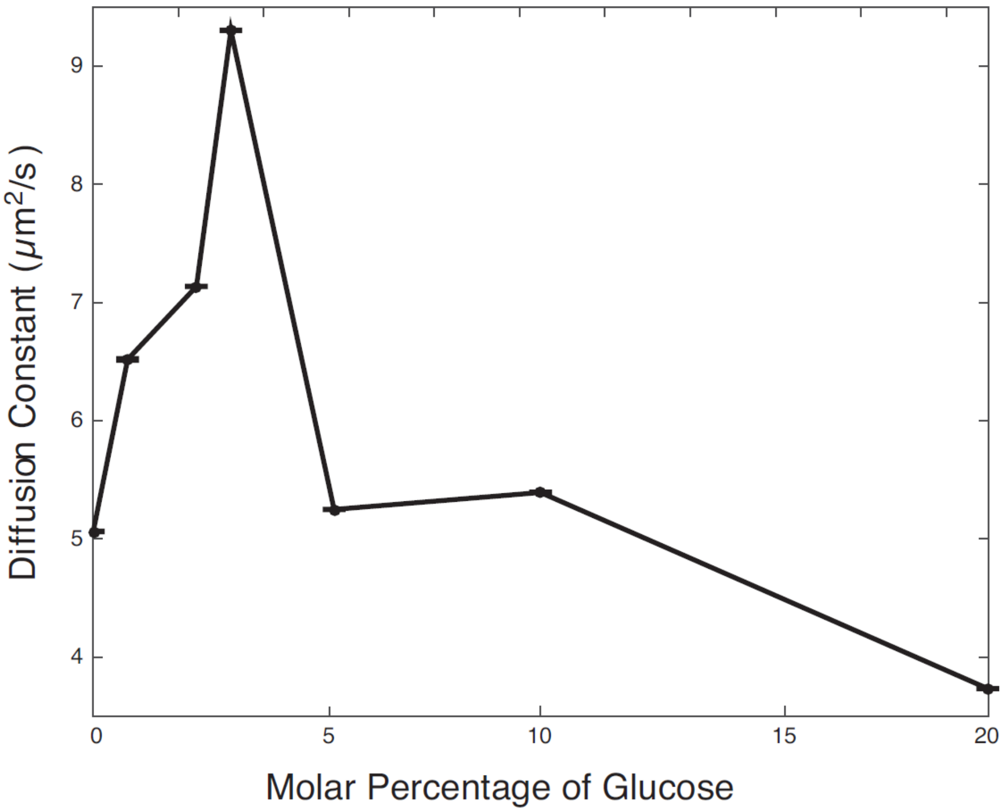

Figure S2. Lateral diffusion constant for phospholipids as a function of molar percentage of glucose from MD simulations. The phosphorous atoms in the lipid headgroups were used to track diffusion and linear regression as the associated mean squared displacements were used to derive a diffusion coefficient. Results correspond well to similarly structured systems [1]. Lipid diffusion peaks at intermediate glucose concentration before again decreasing, achieving values beneath that of a pure dehydrated DMPC bilayer at 20 mol%. The initial increase suggests that the presence of the solute disrupts lipid packing and leads to increased lateral movement at intermediate concentrations; however, at higher glucose levels, the impedence to movement resulting from the additional molecular presence slows down overall movement. This is supported by the hydrogen bonding data wherein the highest sugar concentrations display the lowest asymptotic number of hydrogen bonds per lipid, suggesting that the lipids are not optimally structured due to the increase solute presence. Diffusion constants for glucose molecules were found to be independent of concentration.

Table S1. Parameters of double stretched exponentials used to fit autocorrelation functions for MD systems. All data sets were fit to an equation of the form $f = B + Ae^{(t/T_1)^{\beta_1}} + (A - a)e^{(t/T_2)^{\beta_2}}$. The four methylene carbon windows described here are on the SN1 chain of the DMPC lipids analyzed. All fits achieved an r^2 value greater than 0.99.

Molar Percentage of Glucose (%)	Constant Offset (B)	Atomic Scaling Factor (A)	Atomic Time Constant (T_1)	Atomic Stretching Exponent (β_1)	Molecular Scaling Factor (A–a)	Molecular Time Constant (T_2)	Molecular Stretching Exponent (β_2)
				**Carbon Triplet 4-6**			
0 (Hydrated)	0.0216	0.9	0.2	27.9044	0.6247	499.3788	0.5624
0 (Dehydrated)	0.0298	0.9	0.2	29.3118	0.5893	799.4058	0.5753
0.8 (Dehydrated)	0.0332	0.9	0.2	28.6058	0.5960	724.9174	0.6070
2.5 (Dehydrated)	0.0321	0.9	0.2	29.7790	0.5905	771.3065	0.5963
5 (Dehydrated)	0.0402	0.9	0.2	28.5006	0.5826	816.0912	0.5295
10 (Dehydrated)	0.0348	0.9	0.2	29.6927	0.5877	680.7519	0.5699
20 (Dehydrated)	0.0447	0.9	0.2	28.1668	0.5677	752.8073	0.5782
				**Carbon Triplet 5-7**			
0 (Hydrated)	0.0084	0.9	0.2	23.3853	0.6452	345.5709	0.6211
0 (Dehydrated)	0.0149	0.9	0.2	24.5015	0.6183	533.4344	0.6092
0.8 (Dehydrated)	0.0155	0.9	0.2	24.2215	0.6251	529.0910	0.6492
2.5 (Dehydrated)	0.0127	0.9	0.2	25.3017	0.6198	604.1372	0.6255
5 (Dehydrated)	0.0189	0.9	0.2	24.3347	0.6144	659.6755	0.57
10 (Dehydrated)	0.0163	0.9	0.2	24.7026	0.6184	527.2252	0.65
20 (Dehydrated)	0.0255	0.9	0.2	24.0634	0.5978	681.3700	0.5830
				**Carbon Triplet 6-8**			
0 (Hydrated)	0.0094	0.9	0.1873	20.6085	0.65	244.4182	0.5726
0 (Dehydrated)	0.0152	0.9	0.2	21.4712	0.6297	333.9923	0.5184
0.8 (Dehydrated)	0.0161	0.9	0.2	21.2580	0.6266	334.6376	0.5662
2.5 (Dehydrated)	0.0147	0.9	0.2	21.5870	0.6323	357.1928	0.5675
5 (Dehydrated)	0.0174	0.9	0.2	21.3150	0.6243	395.2746	0.5090
10 (Dehydrated)	0.0156	0.9	0.2	21.5353	0.6301	348.0691	0.5644
20 (Dehydrated)	0.0249	0.9	0.2	20.3496	0.6118	398.8423	0.5547
				**Carbon Triplet 7-9**			
0 (Hydrated)	0.0046	0.9	0.1777	17.1156	0.65	169.0201	0.5793
0 (Dehydrated)	0.0095	0.9	0.2	17.5311	0.6455	206.8435	0.5228
0.8 (Dehydrated)	0.009	0.9	0.2	17.8049	0.6360	196.78	0.5445
2.5 (Dehydrated)	0.0078	0.9	0.2	18.0503	0.6430	218.5694	0.5449
5 (Dehydrated)	0.0096	0.9	0.2	17.9077	0.6349	248.4472	0.4790
10 (Dehydrated)	0.0081	0.9	0.2	18.1400	0.6390	197.6478	0.5306
20 (Dehydrated)	0.0156	0.9	0.2	17.5424	0.6280	261.8248	0.4876

References

1. Rifici, S.; D'Angelo, G.; Crupi, C.; Branca, C.; Conti Nibali, V.; Corsaro, C.; Wanderlingh, U. Influence of Alcohols on the Lateral Diffusion in Phospholipid Membranes. *J. Phys. Chem.* **2016**, *120*, 1285–1290.
